# Occurrence of Endoparasites in Adult Swedish Dogs: A Coprological Investigation

**DOI:** 10.3389/fvets.2021.691853

**Published:** 2021-06-09

**Authors:** Giulio Grandi, Ida Victorsson, Eva Osterman-Lind, Johan Höglund

**Affiliations:** ^1^Section for Parasitology, Department of Biomedical Sciences and Veterinary Public Health, Swedish University of Agricultural Sciences (SLU), Uppsala, Sweden; ^2^Department of Microbiology, National Veterinary Institute (SVA), Uppsala, Sweden; ^3^Blå Stjärnan Animal Hospital, Gothenburg, Sweden

**Keywords:** endoparasites, dog, *Toxocara*, *Giardia*, Sweden

## Abstract

The occurrence of endoparasites in Swedish adult dogs (*n* = 303) was investigated between January and October 2014. Included dogs had to be clinically healthy, older than 1 year and untreated with anthelmintics or endectocides for at least 3 months prior to sampling. They were grouped according to age, category of dog and time since last antiparasitic treatment. Samples were analyzed by flotation to detect parasitic eggs and cysts/oocysts. Among these, 129 (43%) dogs were also analyzed with the Baermann-technique to detect cardiopulmonary larval stages. Parasite dispersal stages were found in 24 (7.9%, CI 95% 4.9–10.1) of the dogs at flotation, while no dog shed cardiopulmonary larval stages. *Giardia* sp. cysts were observed in 2.6% (*n* = 8) of dogs examined, cysts of *Sarcocystis* spp. were observed in 0.6% (*n* = 2), oocysts of *Cystosisopora ohioensis* were found in one dog (0.3%). Eggs of *Toxocara canis* (2.3%, *n* = 7), *Uncinaria stenocephala* (1.3%, *n* = 4) and *Trichuris vulpis* (0.3%, one dog) were found. None of the dogs were diagnosed with more than one species. Although the occurrence of endoparasites was above the average in dogs ≤ 2 years of age (11.5%), nematodes were more common in older dogs ≥4 years (77.0%). Although the occurrence was lower in working/exhibition dogs (5.9%) than in companion dogs (8.4%) and hunting-dogs (8.6%), these differences were not significant. However, dogs exposed to prey according to the owner had a statistically significant higher prevalence than other dogs (20.5 vs. 5.7%). The Odds Ratio (OR) was 4.0 (CI 95%, 1.58–10.11) for dogs having access to prey, 2.4 (CI 95%, 0.37–8.06) for dogs staying at day-care, and 2 (CI 95%, 0.96–5.96) for bitches. Furthermore, a significant association was observed between infection with nematodes and exposures to prey (*p* = 0.006). As a reference, data on the endoparasites in canine fecal samples submitted to the National Veterinary Institute (SVA, Uppsala) during 2014 are presented. Overall, this study shows a low occurrence of endoparasites among dogs in Sweden. Any risk-assessment on zoonotic parasites as well as deworming recommendations will take advantage from these updated figures.

## Introduction

Illegal imports of dogs have increased since Sweden became a member of the European Union in 1995. Also due to changes in legislation, it has also become easier to import dogs for non-commercial purposes (EU Regulation 576/2013). Even though Swedish authorities make their best to prevent introductions of foreign canine parasites, there are no formal requirements for prophylactic antiparasitic treatment for pets traveling into Sweden. This has created opportunities for the introduction of foreign parasites that may establish in the Nordic environment. Sweden represents a particular scenario for parasites because of large/dense populations of wild canids present (i.e., red foxes and wolves) that may act at the same time as a reservoir and as a source of infection to domestic dogs (1, 2). This creates unique conditions favoring the circulation of endoparasites between wild and domestic canids.

When endoparasites were investigated in asymptomatic adult dogs in Sweden in 1999, 17 out of 365 dogs (4.7%) were found to be infected (3). Like in the present study, the dogs recruited were more than 1 year old and they had not been treated with antiparasitics for the least 3 months. Shelters were identified as a major risk factor, while family-owned dogs ran a lower infection risk. A year later a similar study was conducted in Skåne with 230 dogs older than 1 year and 15 (6.5%) were positive for at least one parasite, often *Toxocara canis* (4). The low occurrence of endoparasites in Scandinavian pet dogs was also observed in a Finnish study, where 5.9% (541 dogs examined) of the dogs were infected with *T. canis* (3.1%, *n* = 17) and *Uncinaria stenocephala* (2.6%, *n* = 14) (5). A higher occurrence of endoparasites was recorded in a Danish study, where 22% of hunting dogs (*n* = 178) shed dispersal stages of endoparasites: *T. canis* (12%), *U. stenocephala* (7%), *Taenia* spp. (2%), *Toxascaris leonina* (0.6%), coccidian oocysts (0.6%), trematode eggs (1%) (6). An earlier Danish study performed on companion dogs had showed a lower overall endoparasite prevalence, 3.9%, but these figures could have been biased either by anthelmintic treatments or by the different sampled population (7).

Several studies have shown that patent gastrointestinal parasitic infections in dogs decrease with the age of the dog. For example, in a German study, *T. canis, Cystoisospora* spp. and *Giardia* sp. were more frequently encountered in dogs between 4 and 12 weeks of age than in older dogs (8). In another study of 445 stray dogs from northern Germany nematode eggs and/or oocysts were found in 9% of all dogs (*n* = 42), but 20% amongst those up to 1 year old (9). Not surprisingly, also health status needs to be considered, for example nematode eggs and/or oocysts were identified in 31% of dogs (*n* = 239) from Italian clinics (10). Furthermore, dog management is a factor considered in other studies, for example in Canadian dogs of different ages (*n* = 619), endoparasites were detected in 21% of shelter-sourced dogs and in 15% of homed dogs (11). Although the occurrence was numerically higher in stray dogs than in pet dogs, the difference was not significant. In both groups, however, the highest occurrence was observed in dogs younger than 2 years. The most common parasites were *Giardia* sp., *T. canis* and *Toxascaris leonina*. When zoonotic parasites were investigated in 152 clinically healthy dogs in the Netherlands, the prevalence of *Toxocara* spp., *Giardia* sp. and *Cryptosporidium* spp. was 4, 15, and 9%, respectively (12).

Another important group of endoparasites present in this geographic region is represented by cardiopulmonary metastrongylid nematodes, *Angiostrongylus vasorum* and *Crenosoma vulpis*. *Angiostrongylus vasorum* is well-established in Denmark [where it was most likely imported in 1983 along with a French dog, (13)] in foxes and dogs [prevalence is ranging between 2.2 and 9.7%, (6)], while in Sweden it has been described more recently in the same hosts (in 2003) and is still occurring at a rather low prevalence in dogs (14). In Finland the first autochthonous cases in dogs were described in 2014 and 2017, even if according to a survey among veterinarians other endemic cases of *A. vasorum* occurred earlier (15). *Crenosoma vulpis* is less studied but it is rather common in Danish and Swedish foxes (17.4% and 9% of necropsied foxes harbored it, respectively) (1, 16) as well as in Swedish wolves (39% of wolves harbored metastrongylid larvae negative at *A. vasorum* PCR) (2). *Crenosoma vulpis* was found in 1.4% of Danish dogs showing clinical signs of respiratory and/or circulatory disease [*n* = 4151, (17)], but not in hunting dogs (6). In Swedish dogs, until recently, *C. vulpis* was considered an underdiagnosed parasitic infection; National Veterinary Institute (SVA) had analyzed around 50 samples in 2006 and circa 100 samples in 2008, 12% and circa 20% of them being positive respectively (18). According to available literature, the parasite is well-known by Finnish veterinarians (15). Both *A. vasorum* and *C. vulpis* have been recently found, respectively, in 2.3 and 2.2% of canine fecal samples (*n* = 12,682) collected over several years in Germany (2003–2015). For both parasites, an increase of their prevalence over time was recorded, and for *A. vasorum* based, on the same data, it was even possible to describe its spread in north-eastern Germany (19).

In this study we investigated endoparasites in asymptomatic adult dogs in Sweden through coprological examination. The aim was to update the knowledge about internal parasites in dogs ≥1 year old from households 20 years after becoming members of the EU, with no movement restrictions in place for dogs (i.e., import and travels). This study contributes to and provides a knowledge base for future treatment recommendations of dogs in Sweden. To complete the information provided by our study, we also include results from the coprological analyses performed at the Section for Parasitological Diagnostics at the National Veterinary Institute (SVA) during the same period.

## Materials and Methods

In 2014 fecal samples were obtained from dogs throughout Sweden. Envelopes including sampling instructions and a questionnaire were provided to three animal hospitals (located in Gothenburg, Uppsala and Stockholm) as well as four small animal clinics in Jämtland (Östersund), Halland (Varberg and Veddige) and Stockholm counties. Dog owners visiting those clinics for prophylactic reasons such as vaccinations were then invited to participate in the study. Only two dogs per household were allowed to participate and the inclusion criteria were to be: (i) clinically healthy, (ii) older than 1 year of age, (iii) from households with a maximum of three dogs and (iv) the dogs should not have been treated against parasites for at least 3 months. Dog owners were asked to fill in a questionnaire regarding; (i) gender (female/male), (ii) age (1–2 years, 3–4 years, 5–8 years, or ≥ 9 years), (iii) category of dogs (companion, hunting, working/exhibition, or breeding), (iv) time since last anthelmintic treatment (3–6 months, 6–12 months, ≥ 12 months, or never treated), (v) risk of being exposed to prey (yes/no/do not know), and (vi) if staying at daycare (yes/no).

The owners were instructed to keep their dogs on leash for at least 1 day prior to sampling to prevent coprophagy. Feces should be collected for 3 days, but single samples were also accepted. Samples were then sent to the National Veterinary Institute (SVA) in Uppsala, Sweden and kept refrigerated until the analyses within 7 days.

### Flotation With Centrifugation

Fecal samples (2 g) were thoroughly mixed with 38 ml of 33% solution of ZnSO_4_ (sg 1.17–1.18) using a FILL-FLOTAC container (20). An aliquot of around 15 ml of the mixture was poured to the brim into Clayton-Lane glass tube and placed in a swing-out centrifuge (Sorvall ST40, Thermo Scientific, Gothenburg, Sweden). ZnSO_4_ solution was added until a positive meniscus was observed at the top of the tube, and a coverslip (size 18x18mm) was placed on the top. Tubes were centrifuged for 5 min at 214 × g. After centrifugation the coverslip was removed vertically and placed on a microscopic slide and examined at 100–400 magnification. Parasitic stages (eggs, larvae, cysts and oocysts) were identified according to published keys (21). Results were examined qualitatively and reported as either positive or negative.

### Baermann Examination

Whenever fecal samples collected over 3 days were available from the same dog, they were also analyzed for the presence of nematode larvae of cardiopulmonary nematodes (21). Feces were mixed and a subsample (10 g) was packed in gauze and suspended in a glass funnel. A clamp-sealed plastic tube was placed on the distal end of the funnel and filled with lukewarm tap water and was let to stand for at least 24 h. The clamp was then gently opened and the sediment was drawn off into a glass tube, which was centrifuged for 5 min at 214 × g. The supernatant was removed where after the sediment was transferred to a microscope slide, mixed with a drop of iodine and screened at 40× magnification. Higher magnification, up to 400×, was used for identification of found larvae with the help of published keys (21).

### Data From SVA's Routine Diagnostic Activity

Results from the analyses performed at the National Veterinary Institute (SVA) during the same study period (2014) were collected from an internal database. Samples mainly came from animal hospitals or owners with dogs showing clinical signs. They were analyzed as described above with the difference of protozoan cysts/oocysts of *Giardia* sp. and *Cryptosporidium* sp. that were detected with an immunofluorescent technique (AquaGlo, Waterborne Inc., New Orleans, USA).

### Statistical Analyses

Data was analyzed based on information gathered from the questionnaire. Both total parasite occurrence as well as for nematodes and protozoans separately was calculated with 95% confidence intervals (CI). Risk factors were tentatively identified by studying the associations between occurrence of endoparasites and the considered factors (gender, age, category of dog, time since the last deworming, exposure to prey animals and stay at daycare). Odds ratios (OR) were calculated in Graph Pad Prism (v 6.0) for those factors which seemed to be associated with a higher occurrence (female, hunting, exposure to prey, last anthelmintic treatment ≥ 12 months ago, never treated and staying at daycare). Statistical significance was calculated with Fischer's exact test where *p* < 0.05 was regarded significant.

## Results

### Samples and Response Rates

Fecal samples were received from 303 dogs. Of these, 129 (43%) dogs were collected over 3 days and therefore were also analyzed with a modified Baermann technique for the presence of cardiopulmonary nematodes. The age distribution of the dogs varied between 1 and 16 years, the average age was 5.7 years and the median were 3 years. The distribution among different age classes was the following: 8.7% (*n* = 26) were dogs 1–2 years old, 20.8% (*n* = 62) were dogs 3–4 years old, 41.3% (*n* = 123) were dogs 5–8 years old and 29.2% (*n* = 87) were dogs ≥ 9 years old. The gender distribution was 52% (*n* = 155) female and 48% (*n* = 143) male dogs. The majority of dogs were companion animals (*n* = 227), followed by hunting (*n* = 35) and working/exhibition (*n* = 34). Only one dog was used for breeding. The time span since the latest antiparasitic treatment was classified as follows: (i) within 3–6 months (*n* = 25), (ii) within 6–12 months (*n* = 77), (iii) more than 12 months ago (*n* = 165), or (iv) never dewormed (*n* = 7). According to the owners (i) 212 dogs never ate prey, (ii) 39 dogs had access to prey, and (iii) for 45 it was unknown. Totally 16 dogs were at daycare, while the majority were not (*n* = 277). Response rates to the questions varied between 90.4 and 98.3% (Table 1).

**Table 1:** Response rates from the referral.

**Risk factors**	**Response rate (*n* =)**	**Response rate (%)**
Age	298	98.3
Sex	298	98.3
Latest treatment	274	90.4
Category of dog	297	98.0
Prey	296	97.7
Dogs at daycare	293	96.7

*Total number = 303*.

### Parasite Prevalence and Risk Factors

Dispersal stages of dog endoparasites were detected in 24 (7.9%, CI 95% 4.9–10.1) of the 303 dogs examined (Table 2). Among these, 13 (4.3%, CI 95% 2.0–6.6) were shedding nematode eggs (*T. canis, U. stenocephala, Trichuris vulpis* and *Eucoleus aerophilus*), while 11 (3.6%, CI 95% 1.5–5.7) had protozoan cysts or oocysts (*Giardia* sp., *Sarcocystis* spp., and *Cystoisospora ohioensis*). *T. canis* was the most commonly encountered nematode (2.3% of samples, *n* = 7) while *Giardia* sp. was the most common protozoan (2.6% of samples, *n* = 8). No dog was infected with more than one species and no larvae of cardiopulmonary nematodes were found.

**Table 2:** Occurrence of individual endoparasites.

	**Findings (*n* =)**	**Prevalance (%)**	**95% confidence interval**
**Nematodes**	13	4.3	2–6.6
*Eucoleus aerophilus*	1	0.3	
*Toxocara canis*	7	2.3	
*Uncinaria stenocephala*	4	1,3	
*Trichuris vulpis*	1	0.3	
**Protozoa**	11	3.6	1.5–5.7
*Giardia* sp.	8	2.6	
*Sarcocystis spp*.	2	0.6	
*Cystoisospora ohioensis*	1	0.3	
**Totally**	24	7.9	4.9–10.1

Among the 24 samples containing parasite dispersal stages, 17 were from bitches (70.8%) and 7 from males (29.2%). The occurrence of parasites in infected dogs was highest in the considered youngest group (1–2 years old, *n* = 3/26, 11.5%) and lowest in the oldest ones (≥9 years of age, 6/87, 6.9%) (Table 3). Hunting dogs showed the highest occurrence; 8.6 ± 9.3% (3 of 35), followed by companion dogs; 8.4 ± 3.6% (19 of 227), while the infection level in exhibition dogs was 5.9 ± 11.7% (2 of 34). Only one breeding dog participated in the study and it was negative.

**Table 3:** Total number of findings (*n* =) as well as proportion (%) of nematodes and protozoa per age group.

**Age**	**Numbers investigated**	**Positive**		**Nematodes**		**Protozoa**	
**Years**	***n***	***n***	**%**	***n***	**%**	***n***	**%**
1–2	26	3	11.5	1	3,8	2	7.7
3–4	62	5	8.0	2	3.2	3	4.8
5–8	123	10	8.1	5	4.0	5	4.0
≥9	87	6	6.9	5	5.7	1	1.1
Unknown	5	0	0	0	0	0	0

All dogs dewormed between 3 and 6 months before sampling (*n* = 25) were negative. The infection level in dogs dewormed during the previous 6–12 months was 7.8 ± 5.6% (6 of 77), whereas it was 9.7 ± 4.5% (16 of 165) in those dewormed ≥12 months ago and 14.3 ± 25.9% (1 of 7) in untreated dogs. The occurrence was higher in dogs with access to prey (*n* = 8/39, 20.5%) compared to dogs without access to prey (*n* = 12/212, 5.7%) and with dogs where no information was provided on access to prey (*n* = 4/45, 8.9%). Dogs at daycare seemed to be more often infected (*n* = 2/16, 12.5%) compared to other dogs (*n* = 22/277, 7.9%). The associations (OR) between being infected with endoparasites and potential risk factors are shown in Table 4. Of the examined factors, only exposure to prey animals had statistical significance.

**Table 4:** Odds ratio (OR) for different risk factors.

**Risk factors**	**Infected (*n*)**	**OR ± 95% confidence interval**	***P*-value**
Females	17	2.4 (0.96–5.96)	0.06
Males	7	0.4 (0.17–1.08)	0.09
Companion dogs	19	1.3 (0.47–3.6)	0.81
Hunting dogs	3	1.1 (0.31–3.9)	0.75
Working/exhibition dogs	2	0.7 (0.16–3.13)	1
Dewormed >12 months ago	16	1.7 (0.72–4.21)	0.28
Never dewormed	1	2.0 (0.23–17.15)	0.44
Exposed to prey	8	4.0 (1.58–10.11)	0.006^*^
Staying at day-care	2	1.7 (0.37–8.06)	0.37

* *Significant*.

### Data From Routine SVA's Diagnostic Activity, 2014

During 2014, 5202 analyses were performed in total from sick dogs (no information could be gathered on co-infection status) distributed into 2,301 flotations, 1,817 immunofluorescence tests, and 1,084 Baermann tests. Nematode type eggs of *T. canis, U. stenocephala, Eucoleus* sp. and *T. vulpis* (*n* = 4/2,301) were found in 7.8% (*n* = 180/2,301), 3.6% (*n* = 83/2,301), 1.2% (*n* = 27/2,301) and 0.2% of samples, respectively. Larvae of *C. vulpis* and *A. vasorum* were found in 7.2% (*n* = 78/1,084) and 0.5% (*n* = 5/1,084) of samples, respectively. Protozoan oocysts of *Cystoisospora* spp. were found in 6.9% (*n* = 158/2,301) of the samples at flotation. Cysts of *Giardia* sp. and oocysts of *Cryptosporidium* spp. were detected by immunofluorescence test in 13.4% (*n* = 243/1,817) and 0.2% (*n* = 4/1,817) of samples, respectively.

## Discussion

This study shows that almost 8.0% of the clinically healthy adult dogs in Sweden were infected based on coprological examination. The three most commonly encountered parasites were *Giardia* sp. (2.6%), *T. canis* (2.3%), and *U. stenocephala* (1.3%). Interestingly, the same parasites were found in almost the same proportions in “sick” dogs (data from SVA's diagnostics), but at a higher rates (13, 7.8, and 3.6%, respectively). These findings are in agreement with previous studies on Swedish dogs without clinical signs (3, 4), as well as with those in SVA's diagnostic records. The main difference lies in the detection of *Giardia* sp., but this can be explained by a difference in the diagnostic method used between studies. In the present survey we used 33% ZnSO_4_ solution, which is an optimal flotation medium for *Giardia* cysts in feces (22), while previous Swedish studies were based on saturated NaCl. On the other hand, in samples from sick dogs submitted to SVA routine diagnostics, direct immunofluorescence is used for the detection of *Cryptosporidium* and *Giardia*. This, together with the fact that these pathogens are more frequently found in dogs showing clinical signs, may somehow have contributed to the higher occurrence.

Another difference between the results from clinically healthy dogs and those recorded in SVA's data was the absence of *C. vulpis*, although found in 7.2% of the symptomatic dogs. This supports the view that this pulmonary nematode is sometimes a primary cause of parasitic respiratory distress in Swedish dogs. Furthermore, it is not surprising that no *A. vasorum* was found in asymptomatic dogs, since this parasite is rare in sick dogs; corroborated by data from SVA's diagnostics (0.5%) and from a previous Swedish study (14). Another lungworm that is very common in Danish foxes (74%) is *E. aerophilus* (1). It has been claimed that foxes serve as its reservoir for dogs in the UK (23). Although there is no reason to believe that the situation is so different in Sweden, it was confirmed that the occurrence of *E. aerophilus* was low (1.2%) in sick dogs, whereas it was found in only one asymptomatic 2-year-old bitch which was dewormed ≥12 months ago and that was exposed to prey. This indicates that the overall exposure to *E. aerophilus* is low in Swedish dogs.

Together, our result confirms that endoparasites in adult Swedish dogs still occur at a low level since Sweden became a member of the EU in 1995. Although, a slightly higher prevalence of *T. canis* was previously observed by (3) and (4), but this is probably due to the fact that they also included dogs from multi-dog households, such as kennels. Furthermore, the present results are similar to those coming from other European countries such as Belgium and Netherlands (24), Denmark (7), Finland (5), Germany (25) and the UK (26), showing that between 3.9 and 7.5% of the canine fecal samples were positive for endoparasites. When considering countries with substantially different climatic conditions like Italy, the figures reported for the occurrence of endoparasites were either similar, with 9.7% of the dogs having being found harboring at least one endoparasite (27) or higher (35.3%) but in the latter case the most common helminths detected were *T. vulpis* and hookworms (28) and stray dogs were included in the population. In contrast, both cardiopulmonary and gastrointestinal parasites seem to be more common in central-southern Europe according to recent studies from France (29) and Spain (30), where the overall occurrence of parasite infected dogs were 22% and up to 39%, respectively. Although our figures contribute to give a global indication about parasite occurrence in dogs, a comparison is questionable due to differences in inclusion criteria, sampling strategies and the type of diagnostic methodologies used.

For unknown reasons more female than male dogs were infected with endoparasites in the present study. The proportion of infected females was 11.0%, which is more than two times higher than in males, where only 4.9% were positive. It was expected that the overall occurrence decreased with increasing age. For dogs with nematodes, however, the prevalence of *T. canis* increased among the older animals. It is well-known that patent ascarid infection is common in puppies younger than 6 months (31), but this age group was excluded from the present study. Furthermore, in agreement with Danish studies (6, 7), hunting dogs had a higher occurrence of endoparasites than show dogs in our study, and the most likely explanation is that these had access to prey. This idea was further confirmed by the fact that dogs with access to prey showed a markedly higher occurrence, 20.5%, compared to the no-prey group, 5.7%. The odds ratio for dogs with access to prey was four times higher, and it was the only factor that was significant (*p* < 0.05).

According to a recent survey of pet owners in five European countries, the majority of dogs in Sweden belong to a high risk group for getting exposed and thereby infected with endoparasites (32). In Sweden 500 dog owners participated in the survey, and for a series of behavior/risks (i.e., possibility to go off lead, to have contacts with other dogs, eating prey and/or molluscans, as well as feeding with raw meat) Swedish dogs showed frequencies above the average. Also the percentage of contacts of Swedish dogs with children or elderly people was above the average, raising the issue of an increased risk for zoonotic transmission (32). Although we found a low occurrence of endoparasites in Swedish dogs herein, the risk factors identified by McNamara et al. (32), must be taken into account to perform a more comprehensive risk-assessment. Other recent studies taking in account several risk factors underlined the importance of checking the status of endoparasitic infections in dogs aged between 1 and 7 years old (33) and the rather common unawareness of dog owners regarding the zoonotic risk that their pets could represent (34). Also in our study we observed eggs of *T. canis* only in dogs that were four years old or older, showing that also in Sweden adult clinically healthy dogs can contribute to the environmental contamination. It must be taken in account that ascarid eggs can in fact represent a source of different degrees of contamination of parks and green areas as shown in a recent study (34). Furthermore, the fact that *Giardia* sp. was the most commonly found parasite in both asymptomatic (2.6%) and sick (13.4%) dogs requires an assessment of the risk for zoonotic transmission in the community related to our findings. In humans acute giardia-infection may cause diarrhea, abdominal pain, nausea and weight loss but can also be asymptomatic (35). Nevertheless, among the eight different genotypes of *G. duodenalis* described, only A and B are considered as zoonotic even though the majority of molecular studies have so far not been able to clearly demonstrate transmission from dogs to humans (36). In the present study, no genotyping was conducted. Still, based on our results and on available knowledge, the risk for humans to get infected by zoonotic *Giardia* from clinically healthy dogs in Sweden must be considered as low.

Whether wild canids represent a reservoir of endoparasites for domesticated dogs in Sweden cannot as yet be confirmed since data from most wild carnivores and parasite groups are missing. While an extensive study of helminths of foxes was performed in Denmark (1), no corresponding information is available regarding Swedish foxes where the data is limited to specific parasites like *Echinococcus multilocularis* (37) and *A. vasorum* (14). Similarly, only limited information is available from Swedish wolves, *Canis lupus lupus* (2). In this recent study based on necropsy and coproscopical findings from 20 wolves it was shown that while 90% of them harbored helminths, they were mostly infected by *U. stenocephala* (90%), *Taenia* spp. (45%) and *Eucoleus boehmi* (60%). However, these parasites were absent or rarely occurring in our study. Based on this evidence, it can be hypothesized that in the present scenario the risk of transmission from wild canids to domesticated dogs seems to be low in Sweden.

The low occurrence of endoparasites in asymptomatic adult dogs indicates that the restrictive use of antiparasitic treatments in adult dogs adopted in Sweden—ideally based on the evidence of an active parasitic infection—appears to be effective. By taking advantage of the recently identified risk factors (32) a proper risk assessment for zoonotic transmission could be carried out. One of the missing pieces is to collect more data from wild canids and especially from the red fox (*Vulpes vulpes*) which is the most abundant wild canid in Sweden and the one in closest contact with dogs. The fact that we did not observe any increasing trend between our results and previous studies is suggesting that neither increased import and travel of dogs from Europe to Sweden, nor climate change have caused any evident change in the occurrence of canine endoparasites so far. Overall, this data set represent a baseline for future risk assessment studies and is also a useful tool to assess potential upcoming disturbances in the endoparasitic fauna of dogs.

## Data Availability Statement

The raw data supporting the conclusions of this article will be made available by the authors, without undue reservation.

## Ethics Statement

Ethical review and approval was not required for the animal study because Fecal samples were collected by owners in a non-invasive way. Written informed consent for participation was not obtained from the owners because no invasive procedure was needed.

## Author Contributions

GG, JH, and EO-L planned the study, summarized the results, and took care of writing the article's text until its final version. IV managed the samples' collection, performed the analyses, and wrote a preliminary draft of the article. All authors contributed to the article and approved the submitted version.

## Conflict of Interest

The authors declare that the research was conducted in the absence of any commercial or financial relationships that could be construed as a potential conflict of interest.
